# Association of long-term aspirin use with kidney disease progression

**DOI:** 10.3389/fmed.2023.1283385

**Published:** 2023-12-04

**Authors:** Jun Ling Lu, Prabin Shrestha, Elani Streja, Kamyar Kalantar-Zadeh, Csaba P. Kovesdy

**Affiliations:** ^1^Division of Nephrology, University of Tennessee Health Science Center, Memphis, TN, United States; ^2^VA Connecticut Healthcare System, West Haven VA Medical Center, West Haven, CT, United States; ^3^Harold Simmons Center for Chronic Disease Research and Epidemiology, Division of Nephrology, Hypertension and Transplantation, The Lundquist Institute at Harbor-UCLA Medical Center, Torrance, CA, United States; ^4^Division of Nephrology, Memphis VA Medical Center, Memphis, TN, United States

**Keywords:** chronic kidney disease, microinflammation, aspirin, end-stage kidney disease (ESKD), eGFR slopes

## Abstract

**Background:**

Chronic microinflammation contributes to the progression of chronic kidney disease (CKD). Aspirin (ASA) has been used to treat inflammation for centuries. The effects of long-term low-dose ASA on CKD progression are unclear.

**Methods:**

We examined the association of long-term use of newly initiated low-dose ASA (50–200 mg/day) with all-cause mortality using Cox proportional hazard models; with cardiovascular/cerebrovascular (CV) mortality and with end stage kidney disease (ESKD) using Fine and Gray competing risk regression models; with progression of CKD defined as patients’ eGFR slopes steeper than −5 mL/min/1.73m^2^/year using logistic regression models in a nationwide cohort of US Veterans with incident CKD. Among 831,963 patients, we identified 385,457 who either initiated ASA (*N* = 21,228) within 1 year of CKD diagnosis or never received ASA (*N* = 364,229). We used propensity score matching to account for differences in key characteristics, yielding 29,480 patients (14,740 in each group).

**Results:**

In the matched cohort, over a 4.9-year median follow-up period, 11,846 (40.2%) patients (6,017 vs. 5,829 ASA users vs. non-users) died with 25.8% CV deaths, and 934 (3.2%) patients (476 vs. 458) reached ESKD. ASA users had a higher risk of faster decline of kidney functions, i.e., steeper slopes (OR 1.30 [95%CI: 1.18, 1.44], *p* < 0.01), but did not have apparent benefits on mortality (HR 0.97 [95%CI: 0.94, 1.01], *p* = 0.17), CV mortality (Sub-Hazard Ratio [SHR]1.06 [95%CI: 0.99–1.14], *p* = 0.11), or ESKD (SHR1.00 [95%CI: 0.88, 1.13], *p* = 0.95).

**Conclusion:**

Chronic low-dose ASA use was associated with faster kidney function deterioration, and no association was observed with mortality or risk of ESKD.

## Introduction

In recent years, several studies have uncovered chronic low-grade inflammation as a mechanism behind the progression of many illnesses, such as malignancies (1), diabetes mellitus (2, 3), and cardiovascular diseases (4). Similar inflammatory processes have also been detected in patients with chronic kidney disease (CKD) and those receiving dialysis (5–9) with levels of inflammatory biomarkers, showing an inverse association with the level of kidney function (10). In patients with CKD, chronic inflammation is associated with worse clinical outcomes, such as an increased risk of all-cause mortality and cardiovascular events, development of protein-energy wasting (PEW), or resistance to correction of anemia (11–13). Despite these findings, there is considerable uncertainty about the clinical impact of treatments targeting chronic inflammation in patients with CKD.

Salicylate has been used in the treatment of inflammation for over three centuries. Among the available anti-inflammatory agents, acetylsalicylic acid (ASA), also known as aspirin, has been especially favored due to its long track record, favorable side effect profile, and demonstrated therapeutic effect on many illnesses (14). Although it is well recognized that ASA, at its higher doses, irreversibly inhibits cyclooxygenase isoenzymes (COX), especially COX-1, during acute inflammation processes, the potential of long-term low-dose ASA to achieve anti-inflammatory effects in chronic illnesses has been a matter of debate (15, 16). Multiple potential mechanisms beyond the COX enzymes such as inhibiting the activation of nuclear factor (NF)-kappa B (17), triggering lipoxins (18), or inhibiting Indoleamine 2,3-Dioxygenase (IDO) (19, 20) were investigated. Some studies have successfully shown that low-dose ASA reduces the risk of recurrent colorectal adenomas through anti-inflammatory effects (21). ASA has also been examined for primary prevention of cardiovascular events in patients with diabetes mellitus (22), but its effect on the progression of kidney disease in patients with established CKD has not been well studied. In this large national historic cohort, we examined the association of low-dose long-term ASA therapy with mortality, incident ESKD, and progression of CKD in patients with incident CKD. We hypothesized that ASA therapy is associated with lower mortality and lower risk of kidney end points.

## Methods

### Study population

**Figure 1:**
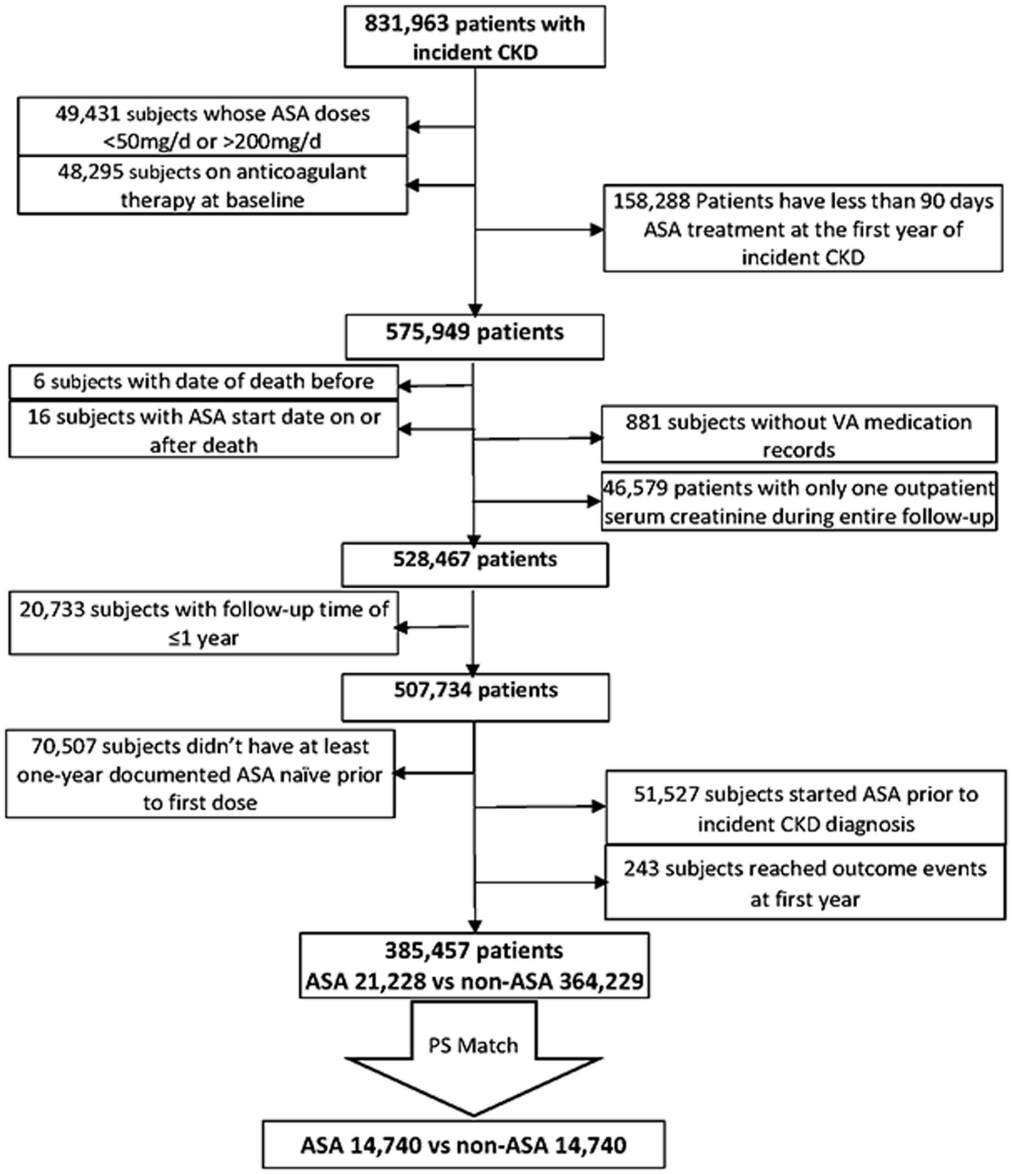
Flowchart of cohort selection.

We used data from the Therapeutic Interventions in Chronic Kidney Disease (TRI-CKD) study (23), a retrospective cohort study which includes 3,562,882 US Veterans who received clinical care in any of the VA healthcare facilities and who had estimated glomerular filtration rate (eGFR) of ≥60 mL/min/1.73 m^2^, recorded from 1 October 2004 to 30 September 2006. eGFR was calculated by using the Chronic Kidney Disease Epidemiology Collaboration (CKD-EPI) equation (24). We identified 831,963 patients who developed CKD after 1 October 2004 based on two outpatient eGFR levels <60 mL/min/1.73m^2^, that were at least 25% lower than the baseline value at cohort entry, and/or two outpatient urine albumin–creatinine ratio (UACR) levels >30 mg/g, both at least 90 days after and within 365 days. The earliest date with eligible values either for eGFR or UACR was used as the incident CKD date. We examined ASA exposure during the first year after the diagnosis of incident CKD. We excluded patients whose ASA dose was >200 mg or < 50 mg/day and whose ASA exposure was less than 90 days during this 1-year evaluation period. We further excluded patients who started ASA treatment outside of the evaluation period, who did not have at least 1 year of ASA-naive period prior to the first dose, or whose follow-up period was less than 1 year. We also excluded patients who received long-term anticoagulation treatments at baseline or who only had a single outpatient eGFR measurement throughout the entire follow-up. Our final study population included 385,457 individuals with incident CKD, among which 21,228 were incident new users of ASA and 364,229 were not exposed to ASA (Figure 1).

Information on baseline demographic characteristics, vital signs, comorbid conditions (defined based on ICD9/ICD10 codes during 2 years prior to the CKD diagnosis) was collected from the VA Corporate Data Warehouse (CDW) (25). Information on prescribed medications was extracted from the outpatient and inpatient pharmacy files of DSS National Data Extracts (26), including the date of dispensation and the dose and the number of pills supplied, and from Medicare Part D files. We also collected information related to outside VA pharmacies from non-VA medication files in CDW including over-the-counter (OTC) medications, herbal supplements, VA-prescribed medications filled at non-VA pharmacies, and medications prescribed by non-VA providers. Baseline blood pressure was defined as the measurement closest to the date of incident CKD performed either within 2 years prior to or during the first 90 days after CKD diagnosis. Laboratory characteristics were obtained from the DSS National Data Extracts Laboratory Results file and the VA LabChem file in the CDW. Baseline medication compliance was assessed based on patients’ adherence to commonly used lipid-lowering agents and antihypertensive drugs (RAAS inhibitor, diuretics, and other antihypertensive drugs) during 1 year prior to incident CKD diagnosis. We defined compliant patients as those who had pharmacy dispensations of the above medications in three out of four calendar quarters, with at least 70% coverage of all days within each quarter. Patients who never received a prescription for lipid lowering and antihypertensive medications were not included in the assessment, and their compliance data were considered as missing.

A propensity score (PS)-matched cohort was created using a 1:1 nearest-neighbor matching without replacement after calculating the propensity scores for the likelihood of presence vs. absence of chronic low-dose ASA by using logistic regression models including age, race, sex, body mass index (BMI), baseline systolic and diastolic blood pressure, eGFR, UACR, smoking, comorbidities (coronary heart disease, cerebrovascular disease, congestive heart failure (CHF), peripheral artery disease, rheumatologic disease, malignancy, liver disease, chronic lung disease, HIV, dementia, paralysis, diabetes, gastrointestinal ulcer or bleeding, and the Deyo-modified Charlson comorbidity index (CCI)), and baseline medication use (renin-angiotensin-aldosterone system (RAAS) inhibitors, statins, insulin, diuretics, non-steroidal anti-inflammatory drugs (NSAIDs), and corticosteroids), and medication compliance. Our final cohort included 14,740 patients in the treated and untreated groups.

### Outcomes

Our co-primary outcomes were all-cause mortality, cardiovascular/cerebrovascular (CV) mortality, and ESKD (defined as initiation of renal replacement therapy (RRT): dialysis or pre-emptive kidney transplant), which were identified from the US Renal Data System (USRDS) (27) and rapid decline of kidney function (defined as the presence of an average decrease (slope) in eGFR of more than 5 mL/min/1.73 m^2^/year). Slopes were calculated from a minimum of two outpatient eGFR values that were at least 180 days apart during the entire follow up period, using a mixed effect model. All-cause mortality was ascertained from the VA Vital Status Files, which record dates of death or dates of last encounter based on all available sources in the VA system (28). Information on cause-specific mortality was obtained from the National Death Index.

### Statistical analysis

Descriptive analyses were performed by using means ± standard deviation (SD), medians (interquartile range, IQR), and proportions as appropriate, and comparisons were made using standardized differences. Follow-up for analyses started 365 days after the diagnosis of incident CKD. Associations of ASA use with all-cause mortality were examined using Cox proportional hazard models; with CV mortality and with ESKD by using Fine and Gray (29) competing risk regression models; and with steeper eGFR slopes using logistic regression models. Patients were followed in survival analyses from the end of the first year after the CKD diagnosis to the first occurrence of death, ESKD, or were censored at the date of the last healthcare service or on 31 December 2018 (the last date for which data on ESKD were available).

Before matching, data points were missing for BMI (0.8%), UACR (16.8%), smoking (18.1%), and baseline compliance (7.3%). After PS matching, no missed data points were observed.

Statistical analyses were performed using Stata MP version 17 (Stata Corporation, College Station, TX) and SAS 9.4 (SAS Institute Inc.; Cary, NC).

## Results

Before PS matching (Supplementary Table S1), the mean baseline age (SD) of ASA users versus non-users was 66.8 (11.1) versus 70.2 (10.9) years old, and White race was less common in ASA users (71.0% versus 83.6% in non-users). ASA users (vs. non-users) also had a higher baseline prevalence of chronic illnesses, such as liver disease (7.3% vs. 4.8%), diabetes mellitus (56.7% vs. 49.0%), gastrointestinal bleeding (6.2% vs. 3.7%), and cerebrovascular disease (13.2% vs. 9.3%) and had a higher baseline UACR (130.8 mg/gm vs. 89.0 mg/gm). Baseline medication use was similar in the two groups, except for insulin (14.1% vs. 8.6%) and NSAID (23.4% vs. 16.4%) use. Overall, medication compliance at the baseline was lower in ASA users (47.8% with good compliance vs. 58.8% in non-users) (Supplementary Table S1). After PS matching, there were no major differences in baseline characteristics (Table 1).

**Table 1:** Baseline characteristics.

		Propensity score matched cohort		
		ASA (14,740)	None (14,740)	Standard difference
Age (yrs)		66.1 ± 10.7	66.2 ± 11.0	<0.01
Gender (%Male)		14,140 (95.9)	14,100 (95.7)	0.01
Race	White	10,520 (71.4)	10,829 (73.5)	0.13
	Black	3,163 (21.5)	2,530 (17.2)	
	Other	1,057 (7.2)	1,381 (9.4)	
Mean SBP (mmHg)		136 ± 22	136 ± 21	<0.01
Mean DBP (mmHg)		77 ± 14	77 ± 13	<0.01
Baseline eGFR (mL/min/1.73 m^2^)		62.1 ± 21.7	62.3 ± 21.1	<0.01
BMI (kg/m^2^)		30.7 ± 6.6	30.7 ± 6.3	<0.01
UACR (mean) (mg/g)		127.3	134.5	0.01
Never smoke		3,333 (22.6)	3,587 (24.3)	0.07
CCI		2.6 ± 2.3	2.6 ± 2.3	0.01
CHF		2,483 (16.8)	2,461 (16.7)	<0.01
MI		1,205 (8.2)	1,182 (8.0)	<0.01
Malignancy		2,315 (15.7)	2,289 (15.5)	<0.01
Liver disease		1,123 (7.6)	1,108 (7.5)	<0.01
Cerebrovascular disease		1,890 (12.8)	1,893 (12.8)	<0.01
Lung disease		3,977 (27.0)	3,935 (26.7)	<0.01
Diabetes		8,848 (60.0)	8,905 (60.4)	<0.01
Peripheral artery disease		2,135 (14.5)	2,103 (14.3)	<0.01
HIV		139 (0.9)	126 (0.9)	<0.01
GI bleeding		891(6.0)	865 (5.9)	<0.01
Peptic ulcer disease		347 (2.4)	330 (2.2)	<0.01
Dementia		663 (4.5)	657 (4.5)	<0.01
Paralysis		351(2.4)	358(2.4)	<0.01
Connective tissue disease		330 (2.2)	326 (2.2)	<0.01
Steroid therapy		440 (3.0)	464 (3.1)	0.03
RAAS inhibitor		9,767 (66.3)	9,704 (65.9)	<0.01
Anti-hypertensive medication		11,621 (78.9)	11,518 (78.1)	0.04
Diuretics		7,046 (47.8)	6,942 (47.1)	0.02
Oral diabetic medication		6,835 (46.4)	6,758 (45.8)	0.03
Insulin		2,288 (15.5)	2,103 (14.3)	0.05
NSAIDs		3,677 (24.9)	3,345 (22.7)	0.08
Cholesterol lowering medications		10,339 (70.1)	10,385 (70.5)	0.07
Compliance		7,358 (49.9)	7,305 (49.6)	<0.01
eGFR slope		−0.9 ± 3.0	−0.8 ± 2.8	0.05

Values expressed as number (percent), mean ± standard deviation, or median (25 percentile, 75 percentile). UACR, urine albumin-creatinine ratio; CCI, Charlson Comorbidity Index; CHF, congestive heart failure; MI, myocardial infarction; BMI, body mass index; eGFR, estimated glomerular filtration rate; SBP, systolic blood pressure; DBP, diastolic blood pressure; GI, gastrointestinal; HIV, human immunodeficiency virus; NSAIDs, Non-steroidal anti-inflammatory drugs; RAAS, renin-angiotensin-aldosterone system.

### Association of ASA treatment with all-cause mortality and CV mortality

In the PS-matched cohort, 6,017 versus 5,829 patients died among ASA users versus non-users, respectively, (event rate: 74.9/1000 patient-years [95%CI: 73.1, 76.8] vs. 76.5/1000 patient-years [95%CI: 74.6, 78.5]) over a median follow-up time of 4.9 years (Table 2). ASA use was associated with a non-significant lower mortality risk (hazard ratio 0.97 [95%CI: 0.94–1.01], Table 3). We detected significant interactions with age, sex, race, and diabetes mellitus, with lower risk of mortality limited to patients who were younger than 65 years old, male, black, or diagnosed with diabetes mellitus (Supplementary Figure S1).

**Table 2:** Event numbers and event rates in the propensity score matched cohort.

	PS matched cohort (*n* = 29,480)			
	*N* = 14,740 in each group			
Event	Number of events		Event rate (events per 1,000 patient-years, 95%CI) or proportion with event (%)	
	ASA use	No ASA use	ASA use	No ASA use
All-cause mortality	6,017	5,829	74.9 (73.1, 76.8)	76.5 (74.6, 78.5)
CV mortality	1,616	1,436	20.1 (19.2, 21.1)	18.9 (17.9, 19.9)
ESRD	476	458	5.9 (5.4, 6.5)	6.0 (5.5, 6.6)
Steep slope	977	761	6.6	5.2

**Table 3:** Associations between ASA and three outcomes in the propensity score matched cohort.

	Hazard ratio/sub-distribution hazard ratio/odds ratio	95%CI	*p*
All-cause mortality	0.97	0.94–1.01	0.17
CV mortality	1.06	0.99–1.14	0.11
ESKD	1.00	0.88–1.13	0.95
Steep slope	1.30	1.18–1.44	<0.01

In an analysis of cause-specific mortality, 1,616 ASA users versus 1,436 non-users died of CV-related causes (event rate: 20.1/1000 patient-years [95%CI: 9.2, 21.1] vs. 18.9/1000 patient-years [95%CI: 17.9, 19.9], respectively) (Table 2). ASA use was associated with marginally higher risk of CV-related death (Sub-Hazard Ratio [SHR] 1.06 [95%CI: 0.99–1.14], Table 3).

### Association of ASA treatment with renal outcomes

The median number of outpatient eGFR values used in slope calculation was 38 versus 22; the mean slope in the PS-matched cohort was −0.9 ± 3.0 versus −0.8 ± 2.8 mL/min/1.73 m^2^/year in ASA users versus non-ASA users, respectively. Faster decline of kidney function occurred in 6.6% versus 5.2% of ASA users versus non-users (Table 2). ASA use was associated with higher risk of rapid deterioration of kidney function; the odds ratio (OR) of steeper slopes in ASA users versus non-users was 1.30 (95%CI: 1.18–1.44), *p* < 0.01 (Table 3). Associations were consistent in subgroup analyses (results not shown).

In the PS-matched cohort, 476 ASA users reached ESKD (event rate: 5.9/1000 patient-years [95%CI: 5.4, 6.5]) compared with 458 non-ASA users (event rate: 6.0/1000 patient-years [95%CI: 5.5, 6.6]). ASA use (vs. non-use) was not associated with ESKD risk (SHR 1.0 [95%CI: 0.88, 1.13], Table 3). We detected significant interactions with age and diabetes mellitus, with a lower risk of ESKD limited to subgroups older than 65 years and to those without diabetes mellitus (Supplementary Figure S1).

## Discussion

In this large national cohort of US veterans with incident CKD, we describe an association of low-dose long-term ASA use with a higher risk of more rapid kidney function deterioration, but there is no apparent benefit on mortality or ESKD. The findings related to all-cause mortality including CV mortality were not in line with the well-known cardiovascular effects of low-dose ASA verified by clinical trials in the 1990s (30), but was consistent with the recent studies (31–33), even though the point estimate of the HR was slightly lower in ASA users in our PS-matched cohort. Earlier clinical trials in patients with preexisting CKD were not beneficial to cardiovascular outcomes, which raised questions about the magnitude of potential cardiovascular effects of ASA in this group of patients (32, 34, 35). Potential reasons for a lack of benefit for CV mortality in our population include residual confounding, and CV mortality is unrelated to occlusive vascular events. A substantial proportion of CV mortality in patients with advanced CKD and ESRD is attributable to sudden cardiac death (36), which, in this population, may not be affected by ASA therapy.

Clinical trials examining the kidney effects of ASA therapy were small or had limited follow-up (33, 37), and hence were unable to provide a conclusive answer about the potential kidney benefits of ASA. A recent post-hoc analysis of a large community-based clinical trial also reported no difference in the slopes of eGFR and UACR among community-dwelling adults with or without CKD treated with 100 mg/day of ASA versus those receiving placebo (38, 39). These findings raised questions about the effectiveness of ASA as the primary prevention for kidney-related outcomes (38, 40), in contrast to other benefits of low-dose ASA therapy such as cancer prevention (16, 41). We examined slopes over an extended period (the median follow-up period was 4.9 years) and in a large cohort of incident CKD patients, which allowed for a robust assessment of long-term kidney effects. The non-significant association of ASA therapy with ESKD did not suggest a long-term benefit (or harm), but ASA therapy was associated with unfavorable eGFR slopes. Our results did not provide clear evidence about potential reno-protective effects versus nephrotoxicity of low-dose ASA. A potential explanation for the result is an acute hemodynamic effect of ASA on kidney function that may affect the calculation of eGFR slopes (42). Further clarification of the long-term kidney effects of ASA on patients with preexisting CKD will require properly powered clinical trials, such as the ongoing LEDA trial (43).

We examined the association of low-dose (50–200 mg/day) chronic ASA exposure (>90 days) with the studied end points. The ideal ASA dose and exposure length remain unclear, especially as it relates to kidney end points. While it is accepted that high-dose ASA can significantly inhibit COXs and all four prostaglandin (PG) isomerases (44), the anti-inflammatory effects of low-dose ASA (especially when applied long term) are less clear. It is also unclear to what extent, benefits of low-dose ASA may be mediated by its anti-inflammatory or antiplatelet effects or a combination of these. The putative mechanism of action mediating the effects of ASA on clinical end points is a combined effect on prostaglandin (PG), nuclear factor erythroid 2-related factor 2 (Nrf2), and/or Interleukin 6 (IL-6) (31, 45, 46) production, along with antiplatelet function, although the relative contribution of each of these mechanisms is uncertain. Previous studies suggested that the anti-inflammatory and antiplatelet effects of ASA may be interrelated, suggesting that both may play a role in clinical outcomes (47, 48). Uremic toxins in progressive CKD patients may enhance inflammation and could induce platelet dysfunction (23), and hence, both physiological effects of ASA may be exerted in this population. The dose of ASA that provides the ideal combined effect and best clinical outcomes in this population remains unclear and requires additional investigation.

Our study has several limitations. This is an observational study in which patients were not randomly assigned to ASA and a matching placebo; therefore, unmeasured characteristics associated with the indication to initiate ASA therapy may have affected the observed outcomes. Most individuals in our cohort were male US veterans and suffered from a high prevalence of comorbid conditions, which limits the generalizability of our findings to women, general population, or individuals from other geographic regions of the world. We used PS matching to balance the characteristics of both groups, but this approach excludes a large number of individuals from the analyses, further limiting external validity. We calculated eGFR slopes using mixed effect model, which assumed a linear change in kidney function over time and did not account for potential non-linear changes in eGFR. Our definition of ASA exposure was limited to patients taking low doses of the drug over an extended time period, and thus, our results may not apply to patients who received different (e.g., higher) doses or experienced different lengths of exposure.

## Conclusion

Chronic low-dose ASA use was not associated with a lower risk of death, and its association with long-term renoprotection is unclear. The long-term kidney effects of ASA therapy on patients with preexisting CKD will need to be clarified in future studies, including clinical trials.

## Data availability statement

The original contributions presented in the study are included in the article/Supplementary material, further inquiries can be directed to the corresponding author.

## Ethics statement

Written informed consent was not obtained from the individual(s) for the publication of any potentially identifiable images or data included in this article because data was obtained from the national VA research database, which contains identifiable information on all US veterans.

## Author contributions

JL: Conceptualization, Data curation, Formal analysis, Investigation, Methodology, Project administration, Supervision, Validation, Writing – original draft, Writing – review & editing. PS: Data curation, Formal analysis, Methodology, Software, Writing – review & editing. ES: Data curation, Formal analysis, Software, Writing – review & editing. KK-Z: Validation, Visualization, Writing – review & editing. CK: Conceptualization, Funding acquisition, Investigation, Methodology, Supervision, Visualization, Writing – review & editing.
