# Supplementary material for: Association of long-term aspirin use with kidney disease progression
Source: Front Med (Lausanne). 2023 Dec 4;10:1283385. doi: 10.3389/fmed.2023.1283385 (PMC10726126; doi:10.3389/fmed.2023.1283385)
Supplement: Supplementary file 1 [file Data_Sheet_1.PDF]

Supplemental Table 1: Baseline Characteristics before propensity score matching

		Un-matched Cohort		
		ASA (21,228)	No ASA (364,229)	Standard Difference
**Age (yrs)**		66.8±11.1	70.2±10.9	0.31
**Gender (%Male)**		20,386 (96.0)	348,432 (95.7)	0.02
**Race**	White	15,066 (71.0)	304,649 (83.6)	0.31
	Black	4,598 (21.7)	42,781 (11.7)	
	other	1,564 (7.4)	16,799 (4.6)	
**Mean SBP (mmHg)**		136±23	134±20	0.08
**Mean DBP (mmHg)**		77±14	75±12	0.16
**Baseline eGFR (mL/min/1.73 m^2)**		60.4±21.1	59.7±19.2	0.07
**BMI (kg/m^2)**		30.5±6.5	30.2±6.2	0.05
**UACR (mg/g)**		130.8	89.0	0.07
**Never Smoke**		3,980 (18.7)	72,657 (19.9)	0.15
**CCI**		2.6±2.3	2.3±2.2	0.14
**CHF**		3,785 (17.8)	50,608 (13.9)	0.11
**MI**		1,792 (8.4)	23,712 (6.5)	0.07
**Malignancy**		3,372 (15.9)	72,007 (19.8)	0.10
**Liver Disease**		1,558 (7.3)	17,616 (4.8)	0.10
**Cerebrovascular Disease**		2,808 (13.2)	33,768 (9.3)	0.13
**Lung Disease**		5,666 (26.7)	87,151 (23.9)	0.06
**Diabetes**		12,024 (56.7)	178,602 (49.0)	0.15
**Peripheral Artery Disease**		3,099 (14.6)	47,852 (13.1)	0.04
**HIV**		222 (1.0)	3,201 (0.9)	0.02
**GI bleeding**		1,317 (6.2)	13,310 (3.7)	0.12
**Peptic ulcer disease**		529 (2.5)	7,837 (2.2)	0.02
**Dementia**		1,080 (5.1)	13,249 (3.6)	0.07
**Paralysis**		495 (2.3)	4,356 (1.2)	0.09
**Connective Tissue Disease**		474 (2.2)	10,423 (2.9)	0.04
**Steroid therapy**		625 (2.9)	8,917 (2.4)	0.05
**RAAS inhibitor**		13,355 (62.9)	206,751 (56.8)	0.12
**Anti-hypertensive medication**		16,149 (76.1)	259,010 (71.1)	0.09
**Diuretics**		9,938 (46.8)	147,407 (40.5)	0.13
**Oral diabetic medications**		8,938 (42.1)	130,646 (35.9)	0.12
**Insulin**		2,993 (14.1)	31,243 (8.6)	0.17
**NSAIDs**		4,957 (23.4)	59,870 (16.4)	0.17
**Cholesterol lowering medications**		14,108 (66.5)	233,958 (64.2)	0.13
**Compliance**		10,152 (47.8)	213,994 (58.8)	0.28
**eGFR slope**		-0.9 ±2.9	-0.7 ±2.3	0.09

Note: Values expressed as number (percent), mean ± Standard Deviation, or median (25 percentile, 75 percentile). UACR: urine albumin-creatinine ratio; CCI: Charlson Comorbidity Index; CHF: congestive heart failure; MI: myocardial infarction; BMI: body mass index; eGFR: estimated glomerular filtration rate; SBP: systolic blood pressure; DBP: diastolic blood pressure. GI: gastrointestinal; HIV: human

immunodeficiency virus; NSAIDs: Non-steroidal anti-inflammatory drugs; RAAS: renin-angiotensin-aldosterone system.

Supplemental Figure 1: subgroups analysis of all-cause mortality and ESKD

**Subgroup**	ESKD Subhazard ratio (95% CI)	ESKD p-value for interaction	All cause mortality Hazard ratio (95% CI)	All cause mortality p-value for interaction
Age		<0.01		<0.01
>55	1.13 (0.88-1.45)		0.91 (0.8-1.03)	
>55 & ≤65yrs	1.23 (1.01-1.49)		0.94 (0.88-1.01)	
>65 & ≤75yrs	0.62 (0.46-0.83)		1.01 (0.94-1.08)	
>75yrs	0.41 (0.23-0.7)		1.09 (1.02-1.16)	
Sex		0.78		0.02
Male	1 (0.88-1.14)		0.97 (0.93-1)	
Female	0.89 (0.39-2)		1.29 (1.01-1.66)	
Race		0.29		0.05
White	1.66 (1.04-2.63)		1.02 (0.98-1.06)	
Black	1.76 (0.87-3.56)		0.92 (0.84-1.02)	
Smoking		0.32		0.9
Never	0.94 (0.72-1.23)		0.99 (0.91-1.07)	
Active	1.07 (0.9-1.26)		0.97 (0.92-1.02)	
Diabetes mellitus		<0.01		<0.01
Yes	1.11 (0.97-1.29)		0.9 (0.86-0.94)	
No	0.65 (0.49-0.87)		1.11 (1.04-1.17)	

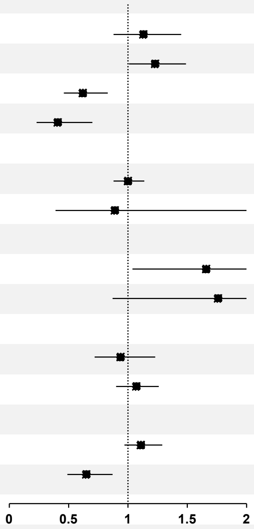

Subhazard ratio (95% CI)

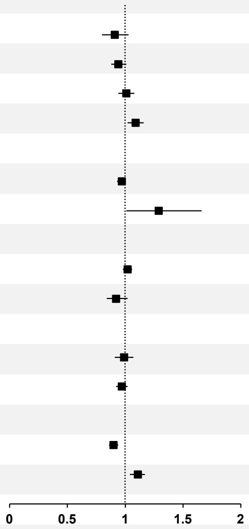

Hazard ratio (95% CI)
